# Editorial: Mental health services for occupational trauma: Decreasing stigma and increasing access

**DOI:** 10.3389/frhs.2022.1041953

**Published:** 2022-11-23

**Authors:** Natalie Mota, Shay-Lee Bolton, Lauren M. Sippel

**Affiliations:** ^1^Department of Clinical Health Psychology, University of Manitoba, Winnipeg, MB, Canada; ^2^Department of Psychiatry, University of Manitoba, Winnipeg, MB, Canada; ^3^Department of Veterans Affairs Northeast Program Evaluation Center, West Haven, CT, United States; ^4^Department of Psychiatry, Geisel School of Medicine at Dartmouth, Hanover, NH, United States; ^5^Department of Veterans Affairs, National Center for Posttraumatic Stress Disorder (PTSD), Washington, DC, United States

**Keywords:** occupational trauma, public safety personnel, healthcare workers, military, mental healthcare

The collection of papers in this Research Topic of *Frontiers Health Services*, “*Mental health services for occupational trauma: Decreasing stigma and increasing access*,” represents innovative international efforts to address the mental health of frontline workers who have dedicated their lives to serving the public in high-risk occupations each and every day. Public safety personnel, military service members, and frontline healthcare workers are likely to be exposed to traumatic events in their lines of work, including physical assaults and sudden deaths (1–3). As cates of mental health problems that frequently occur after trauma exposure, such as depression and posttraumatic stress disorder, are higher among these occupational groups than in the general population (4, 5). The COVID-19 pandemic brought a slew of new challenges that posed further risk to the onset or exacerbation of mental health problems, including high levels of burnout and loneliness (6, 7). Further, barriers to seeking mental healthcare may be more pronounced in these occupational groups. For example, a meta-analysis examining barriers to seeking mental healthcare among first responders identified concerns related to confidentiality and the potential deleterious impact on one's career as common barriers (8). Additional work has noted the important role of stigma, including structural stigma, as an additional barrier in these populations, which can interfere with treatment-seeking through an under-awareness of personal mental health needs and concerns about the perceptions of co-workers about time off work (9–12). Logistical barriers, such as irregular work hours, common to those in these high-risk occupations, may also serve as an additional challenge to accessing mental healthcare.

The time is now then, perhaps more than ever before, to elucidate challenges in accessing mental health services for occupational groups at high risk for trauma exposure. In this Research Topic, the authors undertake this conversation from an international lens, highlighting the extent of the problem in these vulnerable populations and potential ways to better address mental health needs. St. Cyr et al. examined the prevalence and correlates of past-year mental health services use in a nationally representative sample of Canadian military service members and veterans. Roughly a quarter of these individuals sought mental health care, with, as expected, treatment engagement being associated with a mental health diagnosis. The authors also highlighted specific subgroups of individuals, such as those who experienced childhood trauma, sexual assault, and/or recent suicidal ideation, for whom focused efforts to increase help-seeking to mental health services may be most necessary. Reynolds et al. conducted a qualitative study in order to understand experiences of moral injury among workers in long-term care facilities in two Canadian cities. A number of themes emerged when interviewing workers, including the high prevalence of morally injurious experiences while working on the frontlines during COVID-19, the added impact of personal challenges related to the pandemic, and a high prevalence of related mental health difficulties and needs among this sample. The authors hope that these findings can inform the development of effective and tailed supports for this population. Wright et al. present promising results of a virtual stepped care model of mental health resources for first responders that attempts to address a number of potential barriers to care, including perceptions of stigma related to mental health and access to services. Step 1 involves identifying mental health risk *via* self-report screening, which signals the scheduling of a virtual appointment with a clinician in Step 2, and possible recommendations for further treatment following this initial appointment. The authors found preliminary evidence for the utility of their model in connecting first responders to mental health services. From Australia, Pai et al. describe a workplace-based model of wellbeing aimed at reducing burnout, called the SEED Wellness Model, that was developed in a health district for hospital staff during the COVID-19 pandemic. It included a series of implemented initiatives emerging from a person-centered participatory approach, including coffee breaks with a designated “buddy” and a room reserved for quiet reflection. The authors discuss challenges faced in the implementation of the model, including limited resources and staff hesitation. Future directions involve formal and rigorous evaluation of the model to increase generalizability to different healthcare settings. Finally, in their novel experimental study among US Veterans, McGuire et al. examined the role of moral elevation, the positive feelings that can occur after witnessing another's virtuous act, and its role on trauma-related thoughts and emotions hallmark to PTSD. Moral elevation demonstrated promise as a potential clinical intervention that may reduce self-blame, guilt and negative beliefs about the self and others. The ability to shift ways of thinking after exposure to occupational traumas could have a significant and positive impact on development of long-term PTSD consequences in these high-risk populations.

Collectively, these papers highlight the need for mental health services among these vulnerable occupational groups that support the health and wellbeing of the general public, and demonstrate the preliminary feasibility—and challenges—of different treatment models in trying to address the deleterious mental health impacts linked to service. Important directions for future research include identifying strategies to optimize the longer-term uptake of some of these models of care and their effectiveness in the prevention and mitigation of operational stress injuries, as well as their generalizability to a wide range of occupational settings. These investigations represent a critical, collective step forward in understanding how to minimize the impact of work-related mental health sequelae.

## Author contributions

All authors listed have made a substantial, direct, and intellectual contribution to the work and approved it for publication.

## Funding

This study was supported by University of Manitoba, Rady Faculty of Health Sciences Research Start-up Funds (Mota).

## Conflict of interest

The authors declare that the research was conducted in the absence of any commercial or financial relationships that could be construed as a potential conflict of interest.

## Publisher's note

All claims expressed in this article are solely those of the authors and do not necessarily represent those of their affiliated organizations, or those of the publisher, the editors and the reviewers. Any product that may be evaluated in this article, or claim that may be made by its manufacturer, is not guaranteed or endorsed by the publisher.
